# The Cost-Effectiveness of an Intervention Program to Enhance Adherence to Antihypertensive Medication in Comparison With Usual Care in Community Pharmacies

**DOI:** 10.3389/fphar.2019.00210

**Published:** 2019-03-07

**Authors:** Judith E. Bosmans, Danielle M. van der Laan, Yuanhang Yang, Petra J. M. Elders, Christel C. L. M. Boons, Giel Nijpels, Jacqueline G. Hugtenburg

**Affiliations:** ^1^Department of Health Sciences, Faculty of Science, Amsterdam Public Health Research Institute, Vrije Universiteit Amsterdam, Amsterdam, Netherlands; ^2^Department of Clinical Pharmacology and Pharmacy, Amsterdam Public Health Research Institute, Amsterdam UMC, Vrije Universiteit Amsterdam, Amsterdam, Netherlands; ^3^Department of General Practice & Elderly Care Medicine, Amsterdam Public Health Research Institute, Amsterdam UMC, Vrije Universiteit Amsterdam, Amsterdam, Netherlands

**Keywords:** hypertension, medication adherence, antihypertensive medication, cost-effectiveness, community pharmacies, patient-tailored intervention

## Abstract

**Introduction:** Hypertension is considered an important public health issue. Inadequate disease management and non-adherence to antihypertensive medication may result in suboptimal clinical outcomes thereby imposing a financial burden on society. This study evaluates the cost-effectiveness of a patient-tailored, pharmacist-led intervention program aimed to enhance adherence to antihypertensive medication in comparison with usual care.

**Materials and Methods:** An economic evaluation was conducted alongside a pragmatic randomized controlled trial with 9-months follow-up among 170 patients using antihypertensive medication. Effect outcomes included self-reported adherence (MARS-5), beliefs about medicines (BMQ Concern and Necessity scales) and quality-adjusted life-years (QALYs). Costs were measured from a societal perspective. Missing cost and effect data were imputed using multiple imputation. Bootstrapping was used to estimate uncertainty around the cost-differences and the incremental cost-effectiveness ratios. Cost-effectiveness planes and acceptability curves were estimated.

**Results:** There were no significant differences in costs or effects between the intervention program and usual care. The probability of cost-effectiveness of the intervention in comparison with usual care was 0.27 at a willingness-to-pay value of 0 €/unit of effect gained. At a willingness-to-pay value of 20,000 €/unit of effect gained, the probability of cost-effectiveness was 0.70, 0.27, 0.64, 0.87, and 0.36 for the continuous MARS-5 score, dichotomized MARS-5 score, BMQ Concern scale, BMQ Necessity scale and QALYs, respectively.

**Discussion:** In patients with hypertension, the patient-tailored, pharmacist-led intervention program to enhance medication adherence was not considered cost-effective as compared to usual care with regard to self-reported medication adherence, beliefs about medicines and QALYs.

## Introduction

Hypertension is a common chronic condition that is considered an important public health issue worldwide (World Health Organization, 2013). Because of its high prevalence, the costs related to hypertension are substantial (Elliot, 2003; Mennini et al., 2015; Weaver et al., 2015). The financial burden of hypertension is not only related to the treatment of high blood pressure, but also to the costs of care for cardiovascular diseases linked to this condition (Flack et al., 2002; Mennini et al., 2015). Early detection and adequate treatment of hypertension aimed to achieve blood pressure control is important to prevent the onset of cardiovascular diseases. Thus, inadequate management of hypertension and non-adherence to antihypertensive medication are likely to result in suboptimal clinical outcomes (Dragomir et al., 2010; Chowdhury et al., 2013; Kim et al., 2016). In addition, this imposes a substantial economic burden on society in the form of increased health care costs (Sokol et al., 2005; Dragomir et al., 2010; Roebuck et al., 2011) and lost productivity costs due to absenteeism and presentism (Wagner et al., 2012; Unmuessig et al., 2016).

Several pharmacist-led interventions aiming to improve patients’ adherence to (antihypertensive) medication have been developed. In about half of these studies, medication adherence significantly increased, but in only a few studies improved clinical outcomes were observed (Kripalani et al., 2007; Gwadry-Sridhar et al., 2013; Matthes and Albus, 2014). It is also unclear whether costs associated with adverse outcomes related to hypertension and its inadequate treatment are reduced by such interventions.

Research indicates that to effectively improve medication adherence, it is important to develop interventions that use a theoretical framework addressing the complexities of adherence behavior (van Dulmen et al., 2007). With respect to interventions themselves, it is important to consider the preferences and needs of individual patients and incorporate a patient-tailored approach that identifies each patient’s specific barriers to adhere. The CATI study was developed with these specific points in mind (van der Laan et al., 2017).

When implementing new interventions in health care, it should be evaluated whether implementation of the intervention constitutes an efficient use of scarce health care resources (Petrou and Gray, 2011). Evidence on the cost-effectiveness of adherence-enhancing interventions is mixed. A recent review concluded that community pharmacist-led interventions aimed to improve adherence are either cost-saving or cost-effective (Simon-Tuval et al., 2016). However, studies specifically conducted in patients with hypertension indicate that adherence-enhancing interventions are not cost-effective as compared to usual care (Schroeder et al., 2005; Brunenberg et al., 2007).

We have previously shown that the CATI intervention program did not influence self-reported medication adherence, quality of life, illness perceptions or blood pressure. However, participants in the intervention group had stronger beliefs in the necessity of using their medicines after 9 months as compared to the control group (van der Laan et al., 2018). The aim of the present study was to evaluate the cost-effectiveness of the CATI intervention program in comparison with usual care in patients using antihypertensive medication from a societal perspective.

## Materials and Methods

### Study Design

An economic evaluation was conducted alongside a pragmatic randomized controlled trial (RCT) with 9-months of follow-up comparing the CATI intervention program with usual care (van der Laan et al., 2017). The Medical Ethics Committee of the VU University Medical Center approved the study. All participants gave their written informed consent. The study was performed in accordance with the declaration of Helsinki (2008) and the Dutch Medical Research involving Human Subjects Act (WMO). The trial was registered in the Dutch Trial Register (NTR5017).

### Study Population

Twenty community pharmacies from different regions in the Netherlands participated in the study. Patients (45–75 years) who used antihypertensive medication (beta-blockers, calcium antagonists, diuretics, angiotensin converting enzyme (ACE) inhibitors and angiotensin II receptor antagonists) and indicated to have hypertension by self-report were eligible for the trial. Thus, no formal diagnosis of hypertension from a physician was required. Moreover, patients should be considered non-adherent based on both pharmacy dispensing data (Proportion of Days Covered, PDC <80% over the past 6 months) and a self-report questionnaire (Medication Adherence Report Scale, MARS-5 <25 points) (Horne, 2013) to be eligible to participate. Exclusion criteria were insufficient Dutch language skills and the use of repeat dispensing and pill packaging services provided by the pharmacy. Because this was a pragmatic trial that aimed to resemble actual practice as much as possible, there were no additional inclusion and exclusion criteria. Patients willing to participate were randomly assigned by a member of the research team to the intervention or control group using a 1:1 allocation ratio.

### Intervention Group

Participants in the intervention group received the patient-tailored, pharmacist-led CATI intervention program to enhance adherence to antihypertensive medication in addition to usual care. Details of the intervention program have been described elsewhere (van der Laan et al., 2017). In short, the CATI intervention program consisted of two consultations in the pharmacy. During the first consultation, possible barriers to adhere to medication were identified and tailored information and recommendations were provided to overcome these barriers. Each participant was given a written summary to facilitate implementation of the adherence measures agreed upon. After 2–3 months, a follow-up consultation was planned to discuss participants’ implementation of and experiences with treatment, information and advice.

### Control Group

Participants in the control group were given usual care according to the Dutch guidelines of the Royal Dutch Pharmacists Association (2006). These guidelines cover the checking and dispensing of prescribed medication, the provision of instructions on medication use, and informing patients of intended effects and possible side effects, at the first and second dispensing of medication.

### Effect Outcomes

The primary outcome was self-reported medication adherence as measured with the MARS-5. The MARS-5 addresses both intentional non-adherence (four statements: ‘I alter the dose of my medicines,’ ‘I stop taking my medicines for a while,’ I decide to miss out on a dose of my medicines,’ and ‘I take less of my medicines than instructed’) and unintentional non-adherence (one statement: ‘I forget to take my medicines’). Each statement is rated on a five-point scale, from 1 (always) to 5 (never) (Cohen et al., 2009; Mora et al., 2011; Bäck et al., 2012; Salt et al., 2012; Horne, 2013; Lin et al., 2018). The MARS-5 sum score was calculated, ranging from 5 to 25 points where a higher score indicates better adherence. In addition, the MARS-5 sum score was dichotomized at 9-months follow-up, where a score of 25 points indicated that a participant was adherent to medication and a score of 24 points or less that a participant was non-adherent to medication (George et al., 2005; McAdam-Marx et al., 2014; Sandy and Connor, 2015).

Secondary outcomes included participants’ beliefs about medicines and quality of life. Participants’ beliefs about medicines were measured with the Specific Beliefs about Medicines Questionnaire (BMQ Specific) (Horne and Weinman, 1999; Horne et al., 1999; Mahler et al., 2012). The measurement of patient’s beliefs about medication is important because it provides insight into the mechanism by which medication beliefs might influence medication adherence. The BMQ consists of ten questions scored on a 5-point Likert scale and is subdivided into a Concern scale and a Necessity scale, both ranging from 5 to 25 points. The BMQ Specific Concern measures the patients’ concerns about taking medication; a higher score on the BMQ Concern scale indicates less trust in the positive effects of the medication, meaning more concerns. The BMQ Specific Necessity measures the patients’ beliefs of the necessity of taking the medication with a higher score indicating stronger beliefs in the necessity of using the medication. Quality-adjusted life-years (QALYs) were calculated using participants’ quality of life as assessed with the EuroQol (EQ-5D-5L) questionnaire (Hurst et al., 1997; Herdman et al., 2011). The EQ-5D-5L contains five health dimensions rated on a 5-level severity scale. Health states were converted into utility scores using the Dutch tariff (Versteegh et al., 2016). QALYs were calculated by multiplying the utility of a particular health state with the time spent in that health state using the area-under-the-curve method.

### Cost Outcomes

Costs were measured from a societal perspective and included intervention costs, health care costs and lost productivity costs. Costs were measured with adapted versions of the iMTA Cost Questionnaire (iMCQ) (Bouwmans et al., 2013a) and the iMTA Productivity Cost Questionnaire (iPCQ) (Bouwmans et al., 2013b) at 3, 6, and 9 months after baseline. All costs were converted to Euros 2016 using consumer price indices since most data was collected in that year (Statistics Netherlands, 2017).

Intervention costs were estimated using a bottom-up approach. The consultation time for both intervention consultations was determined and subsequently valued using salary information from the collective employment agreement of community pharmacists from the Royal Dutch Pharmacists Association (2016).

Health care costs included use of primary health care (such as visits to the general practitioner, physical or occupational therapist, psychologist or a social worker), secondary health care (such as hospital stays and outpatient clinic visits), supportive home care (such as informal care and professional home care) and the use of prescribed medication. Primary and secondary health care, and supportive home care were valued using Dutch standard costs (Hakkaart-van Roijen et al., 2015). Medication use including use of antihypertensive drugs was assessed using pharmacy dispensing records and was valued using unit prices of the National Health Care Institute (2016). Prices were specific for the brand and dosage of the drugs used.

Lost productivity costs included costs related to work absenteeism, presentism and absenteeism from unpaid work. Absenteeism was valued using the friction cost approach (Koopmanschap et al., 1995) based on gender-specific price weights for work hours lost (Hakkaart-van Roijen et al., 2015). The friction cost approach assumes that a sick employee is replaced after a certain amount of time, the friction period (84 days), after which no further lost productivity costs occur. Presentism concerns hours that participants performed sub-optimally at work because of health complaints and was valued using gender-specific price weights for work hours lost (Hakkaart-van Roijen et al., 2015). Absenteeism from unpaid work concerned hours that participants were unable to perform voluntary work or domestic activities and was valued using a shadow price of €14.13 per hour (Hakkaart-van Roijen et al., 2015).

### Statistical Analysis

Participant characteristics were described using descriptive statistics and included gender, age, origin, educational level (low means no education to elementary education; moderate means preparatory middle-level to middle-level applied education; high means higher general continued education to scientific education) living situation, employment status, tobacco and alcohol use, number of cardiovascular diseases and antihypertensive medicines, comorbidities (such as diabetes, depression, asthma, chronic obstructive pulmonary disease, or rheumatoid arthritis), assistance with medication use, specialist visits and medication review past year, and general satisfaction with medication. The main analyses were conducted according to the ‘intention-to-treat’ principle. Missing cost and effect data were imputed using Multiple Imputation by Chained Equations (MICE) (van Buuren and Groothuis-Oudshoorn, 2011). The imputation model included: (1) all outcomes included in the analysis models, (2) baseline variables that differed between groups, (3) baseline variables that differed between participants with complete and incomplete data, (4) pre-selected confounders (age, gender, and education level), and (5) baseline variables related to the cost and effect outcomes. Predictive Mean Matching was used in the MICE procedure to account for the skewed distribution of costs and fifteen complete datasets were generated to reach a loss of efficiency smaller than 5% (White et al., 2011). All datasets were analyzed separately and the results were pooled according to Rubin’s rules (Rubin, 1987).

For the continuous MARS-5 sum score and the BMQ Concern and Necessity scores, the overall effect over time was estimated using linear mixed model analyses adjusted for the baseline value of the outcome. Differences in QALYs, the proportion of adherent participants based on the dichotomized MARS-5 score and costs were estimated using linear regression analyses. Incremental Cost-Effectiveness Ratios (ICERs) were calculated by dividing the mean difference in total societal costs between the study groups with the mean difference in effects. Bias-corrected and accelerated bootstrapping with 5,000 replications was used to estimate 95% confidence intervals around cost and effect differences, and to estimate the uncertainty surrounding the ICERs which was graphically presented on a cost-effectiveness plane (Black, 1990). Cost-effectiveness acceptability (CEA) curves were estimated in which the probability that the intervention program is cost-effective in comparison with the control group is plotted on the *y*-axis, while the willingness-to-pay (WTP) per incremental unit of effect is plotted on the *x*-axis (Fenwick et al., 2004).

Several analyses were performed. In the main analyses, both costs and effects were adjusted for the baseline MARS-5 sum score. In the first sensitivity analysis, outcomes were additionally adjusted for the potential confounders age, gender and education level. The second sensitivity analysis concerned a ‘per protocol’ analysis adjusted for MARS-5 sum score at baseline. In this analysis only intervention group participants that attended both the first and follow-up consultation at the pharmacy were included. A ‘per protocol’ analysis with adjustment for the potential confounders age, gender and education level was the third sensitivity analysis. Finally, a subgroup analysis was performed among participants who at baseline had a MARS-5 score of 23 or lower (Mårdby et al., 2007; Sjölander et al., 2013), which indicates that they were more non-adherent to their medication. Analyses were performed with SPSS version 22 (IBM Corp, Armonk, NY, United States) and Stata/SE version 14.1 (College Station, TX, StataCorp LP). A *p*-value ≤ 0.05 was considered statistically significant.

## Results

### Participants

In total, 170 patients using antihypertensive medication participated. Of them, 85 were randomized to the intervention group and 85 to the control group. At baseline, the MARS-5 sum score was 0.9 points higher in the control group as compared to the intervention group, which indicates that participants in the intervention group were less adherent than participants in the control group. No other relevant differences in participants’ characteristics were found between the groups (Table 1). Complete data were obtained from 135 participants (79.4%) on the cost/effect outcomes. There were no differences between participants with and without complete follow-up. Of the 85 participants in the intervention group, 66 participants (77.6%) attended both intervention program consultations.

**Table 1:** Baseline results of the intervention group and control group.

Baseline results	Intervention group *n* = 85	Control group *n* = 85
	***n* (%) or mean ± SD**	***n* (%) or mean ± SD**

**Participant characteristics**		
Female gender	44 (51.8)	42 (49.4)
Age in years	60.3 ± 7.9	61.9 ± 7.9
Origin		
Dutch native	78 (91.8)	73 (85.9)
Western immigrant	3 (3.5)	6 (7.1)
Non-western immigrant	4 (4.7)	6 (7.1)
Education level		
Low	23 (27.1)	20 (23.5)
Moderate	31 (36.5)	35 (41.2)
High	31 (36.5)	30 (35.3)
Living situation, alone	10 (11.8)	18 (21.2)
Employment status, working	38 (44.7)	34 (40.0)
Tobacco use, yes	6 (7.1)	9 (10.6)
Alcohol use, yes	59 (69.4)	59 (69.4)
**Disease and medication characteristics**		
Number of cardiovascular diseases	1.9 ± 1.1	1.9 ± 0.9
Comorbidities, yes	44 (51.8)	33 (38.8)
Assistance with medication use, yes	6 (7.1)	4 (4.7)
Number of antihypertensive medicines	1.8 ± 0.8	1.8 ± 0.8
Specialist visit past year, yes	35 (41.2)	32 (37.6)
Medication review past year, yes	16 (18.8)	6 (7.1)
General satisfaction with medication		
Very satisfied	20 (23.5)	21 (24.7)
Fairly satisfied	41 (48.2)	52 (61.2)
Neither satisfied nor dissatisfied	20 (23.5)	12 (14.1)
Fairly dissatisfied	3 (3.5)	0 (0)
Very dissatisfied	1 (1.2)	0 (0)
**Other characteristics**		
MARS-5 sum score	21.6 ± 3.1	22.5 ± 2.2
BMQ concern	12.9 ± 3.7	12.3 ± 3.7
BMQ necessity	15.6 ± 4.4	15.7 ± 4.1
Utility score	0.83 ± 0.2	0.82 ± 0.2

*BMQ, Specific Beliefs about Medicines Questionnaire; MARS-5, Medication Adherence Report Scale; SD, standard deviation.*

### Effects

The effect outcomes after 9 months are presented in Table 2. There were no statistically significant differences in any of the effect outcomes between the groups, both without and with adjustment for potential confounders.

**Table 2:** Multiple imputed effect outcomes and costs after 9 months.

Outcome	Intervention Mean (*SE*)	Control Mean (*SE*)	Crude Δ (95% CI)	Adjusted Δ (95% CI)^∗^	Fully adjusted Δ (95% CI)†
**Effect outcomes**					
MARS sum score			0.20 (-0.35; 0.77)	0.21 (-0.32; 0.74)	0.23 (-0.32; 0.77)
T0	21.6 (0.34)	22.5 (0.24)	NA		
T1	22.4 (0.29)	22.8 (0.26)	NA		
T2	22.7 (0.31)	22.8 (0.30)	NA		
T3	22.6 (0.38)	23.0 (0.30)	NA		
MARS dichotomous	0.33 (0.05)	0.35 (0.05)	-0.02 (-0.17; 0.12)	-0.01 (-0.15; 0.14)	0.004 (-0.14; 0.15)
BMQ concern			-0.19 (-1.1; 0.71)	-0.21 (-1.1; 0.68)	-0.21 (-1.1; 0.70)
T0	12.9 (0.41)	12.3 (0.40)	NA		
T1	13.0 (0.45)	12.8 (0.53)	NA		
T2	12.5 (0.47)	12.3 (0.54)	NA		
T3	12.6 (0.60)	12.3 (0.57)	NA		
BMQ necessity			0.59 (-0.32; 1.5)	0.58 (-0.34; 1.5)	0.58 (-0.35; 1.5)
T0	15.6 (0.47)	15.7 (0.44)	NA		
T1	15.7 (0.49)	15.7 (0.54)	NA		
T2	16.1 (0.59)	16.1 (0.58)	NA		
T3	16.9 (0.68)	15.6 (0.58)	NA		
QALYs	0.62 (0.02)	0.61 (0.02)	0.01 (-0.04; 0.06)	0.02 (-0.03; 0.07)	0.02 (-0.03; 0.07)
**Costs**					
Total healthcare costs	3529 (793)	2535 (634)	994 (-709; 2810)	909 (-754; 2782)	915 (-796; 2844)
Primary care	399 (70)	466 (95)	-67 (-262; 113)	-80 (-277; 100)	-88 (-287; 96)
Home care	704 (266)	692 (329)	11 (-725; 728)	28 (-665; 832)	26 (-685; 854)
Secondary care	2270 (658)	1238 (350)	1031 (-58; 2434)	943 (-126; 2351)	956 (-165; 2421)
Medication	139 (15)	156 (22)	18 (-32; 68)	17 (-32; 70)	20 (-31; 73)
Intervention	48 (3)	0 (0)	NA	NA	NA
Total lost productivity costs	2802 (803)	2572 (740)	229 (-1535; 2021)	76 (-1696; 1900)	-67 (-1924; 1803)
Presentism	1089 (441)	667 (268)	422 (-292; 1575)	366 (-390; 1616)	325 (-468; 1552)
Absenteeism paid work	1147 (488)	1300 (427)	-153 (-1121; 831)	-193 (-1171; 808)	-267 (-1243; 748)
Absenteeism unpaid work	566 (188)	605 (231)	-39 (-563; 419)	-98 (-622; 334)	-125 (-693; 321)
Total societal costs	6379 (1276)	5107 (1123)	1271 (-1535; 4388)	1033 (-1778; 4244)	896 (-2024; 4188)

*SE, standard error; Δ, difference between CATI intervention group and control group; CI, confidence interval; MARS, Medication Adherence Report Scale; NA, not applicable; BMQ, Specific Beliefs about Medicines Questionnaire; QALY, Quality-Adjusted Life-Years. ^∗^ adjusted for baseline MARS-5 sum score; ^†^ adjusted for baseline MARS-5 sum score, age, gender and education level.*

### Costs

Table 2 presents the mean costs and both the crude and adjusted differences in costs between the intervention and control group. The mean costs of providing the complete intervention program (i.e., two consultations with participants) were estimated to be €48 per participant. Health care costs and lost productivity costs were higher in the intervention group compared to the control group, but these differences were not statistically significant. The adjusted difference in total societal costs between the intervention group and the control group was €1033, but this difference was not statistically significant.

### Cost-Effectiveness

For the continuous MARS-5 sum score, an ICER of 4949 was found indicating that one point of improvement in MARS-5 score over time extra in the intervention group as compared to the control group was associated with a societal cost of €4949 (Table 3). Based on the CEA curve, the probability that the intervention program was considered cost-effective compared to the control group was 0.27 when the WTP is 0 €/point of improvement extra, and it increased to 0.70 when the WTP increases to 20,000 €/point of improvement extra. For the dichotomized MARS-5 score, an ICER of -149526 was found indicating that society should be willing to pay €149,526 per adherent participant less (Table 3). The CEA curve indicated that the probability of the intervention program being cost-effective in comparison to the control group was 0.27 when society is not willing to pay anything. This probability decreased to 0.25 at a WTP of 10,000 €/adherent participant extra and then increased again to 0.27 at a WTP of 20,000 €/adherent participant extra.

**Table 3:** Cost-effectiveness outcomes after 9 months.

Outcome	ΔC (95% CI)	ΔE (95% CI)	ICER	CE plane				CEA curve	
				NE	SE	SW	NW	WTP = 0	WTP = 20,000
**Main analysis^∗^**	
MARS sum score	1033 (-1778; 4244)	0.21 (-0.32; 0.74)	4949	56%	22%	5%	17%	0.27	0.70
MARS dichotomous	1033 (-1778; 4244)	-0.01 (-0.15; 0.14)	-149526	38%	8%	18%	36%	0.27	0.27
BMQ concern	1033 (-1778; 4244)	-0.21 (-1.1; 0.68)	-4823	49%	19%	8%	24%	0.27	0.64
BMQ necessity	1033 (-1778; 4244)	0.58 (-0.34; 1.50)	1787	66%	23%	4%	7%	0.27	0.87
QALYs	1033 (-1778; 4244)	0.02 (-0.03; 0.07)	59979	52%	24%	3%	21%	0.27	0.36
**Fully adjusted analysis^†^**	
MARS sum score	896 (-2024; 4188)	0.23 (-0.32; 0.77)	3940	54%	26%	5%	15%	0.30	0.73
MARS dichotomous	896 (-2024; 4188)	0.004 (-0.14; 0.15)	-27994	30%	22%	30%	18%	0.30	0.33
BMQ concern	896 (-2024; 4188)	-0.21 (-1.1; 0.70)	-4301	46%	21%	9%	23%	0.30	0.64
BMQ necessity	896 (-2024; 4188)	0.58 (-0.35; 1.50)	1542	63%	26%	4%	7%	0.30	0.87
QALYs	896 (-2024; 4188)	0.02 (-0.03; 0.07)	47438	50%	27%	3%	19%	0.30	0.40
**Per protocol analysis^∗^**	
MARS sum score	569 (-2411; 3738)	0.13 (-0.39; 0.66)	4262	41%	27%	10%	22%	0.37	0.64
MARS dichotomous	569 (-2411; 3738)	-0.06 (-0.21; 0.08)	5573	12%	8%	49%	31%	0.37	0.18
BMQ concern	569 (-2411; 3738)	-0.04 (-0.99; 0.91)	-15697	33%	19%	17%	30%	0.37	0.51
BMQ necessity	569 (-2411; 3738)	0.50 (-0.43; 1.40)	1136	55%	31%	6%	8%	0.37	0.84
QALYs	569 (-2411; 3738)	0.02 (-0.03; 0.07)	30374	43%	34%	4%	19%	0.37	0.46
**Fully adjusted per protocol analysis^†^**	
MARS sum score	312 (-2820; 3570)	0.16 (-0.39; 0.70)	1963	38%	32%	11%	19%	0.43	0.68
MARS dichotomous	312 (-2820; 3570)	-0.05 (-0.19; 0.10)	15945	14%	14%	49%	23%	0.43	0.27
BMQ concern	312 (-2820; 3570)	-0.02 (-0.98; 0.95)	-19833	29%	22%	21%	28%	0.43	0.50
BMQ necessity	312 (-2820; 3570)	0.50 (-0.44; 1.50)	617	50%	35%	7%	7%	0.43	0.85
QALYs	312 (-2820; 3570)	0.02 (-0.03; 0.08)	14442	40%	39%	4%	17%	0.43	0.52
**Subgroup analysis^#^**	
MARS sum score	201 (-3672; 3864)	0.51 (-0.28; 1.3)	391	49%	41%	5%	5%	0.46	0.88
MARS dichotomous	201 (-3672; 3864)	0.07 (-0.11; 0.25)	2799	47%	32%	14%	7%	0.46	0.70
BMQ concern	201 (-3672; 3864)	-0.11 (-1.4; 1.20)	-1900	29%	27%	18%	25%	0.46	0.56
BMQ necessity	201 (-3672; 3864)	0.58 (-0.73; 1.90)	343	46%	36%	10%	8%	0.46	0.81
QALYs	201 (-3672; 3864)	0.04 (-0.03; 0.10)	5598	45%	41%	5%	9%	0.46	0.58

*ΔC, cost difference between CATI group and control group; ΔE, effect difference between CATI group and control group; CI, confidence interval; ICER, Incremental Cost-Effectiveness Ratio; CE plane, cost-effectiveness plane; CEA curve, cost-effectiveness acceptability curve; NE, northeast (more expensive and more effective); SE, southeast (less expensive and more effective); SW, southwest (less expensive and less effective); NW, northwest (more expensive and less effective); WTP, willingness-to-pay; MARS-5, Medication Adherence Report Scale; NA, not applicable; BMQ, Specific Beliefs about Medicines Questionnaire; QALY, Quality-Adjusted Life-Years. ^∗^ adjusted for baseline MARS-5 sum score; ^†^ adjusted for baseline MARS-5 sum score, age, gender, and education level; ^#^ baseline MARS-5 sum score lower than 23.*

For the BMQ Concern scale, the ICER was -4823 indicating that to obtain one point of improvement in BMQ Concern score an investment of €4823 is needed. For the BMQ Necessity scale, the ICER was 1787, indicating that one point of improvement in BMQ Necessity score is associated with an investment of €1787. For both BMQ scales, the CEA curve gradually increased from 0.27 at a WTP of 0 €/point improvement to 0.64 for the BMQ Concern scale and 0.87 for the BMQ Necessity scale at a WTP of 20,000 €/point improvement.

The ICER for QALYs was 59979, indicating that societal costs in the intervention group were on average €59979 higher than in the control group per QALY gained (Table 3). The CEA curve showed that the probability of the intervention program being cost-effective was 0.27 when society is not willing to pay anything per QALY gained. This probability gradually increased to 0.36 at a willingness-to-pay of 20,000 €/QALY.

### Sensitivity Analyses

In the fully adjusted analysis (Table 3), the difference in total societal costs became somewhat smaller, but costs in the intervention group were still non-significantly higher than in the control group. The estimated effect differences were similar to the main analysis. The probability that the intervention program is cost-effective in comparison with usual care was slightly higher than in the main analysis, but the overall results did not change.

In the ‘per protocol’ analyses (Table 3), the differences in total societal costs decreased as compared to the main and fully adjusted analyses, but these differences were not statistically significant. The effectiveness of the intervention program as compared to the control group was similar to the main analysis. Again, the probability of cost-effectiveness was higher than in the main analysis, but this did not affect the overall results.

The difference in total societal costs in the *post hoc* subgroup analysis was smaller than in the main analysis and effects were slightly larger. This resulted in smaller ICERs as compared to the main analysis and higher probabilities of cost-effectiveness of the intervention program in comparison with control for all outcome measures.

## Discussion

The aim of the current paper was to evaluate the cost-effectiveness of the CATI intervention program to enhance adherence to antihypertensive medication as compared to usual care from a societal perspective. There were small, statistically non-significant improvements in adherence-related behavior, patients’ beliefs about medicines, and quality-adjusted life-years in the intervention group as compared to the control group. Total societal costs in the intervention group were statistically non-significantly higher than in the control group. The cost-effectiveness analysis showed that the CATI intervention program was not cost-effective in comparison with usual care.

Two recent systematic reviews examined the cost-effectiveness of adherence-enhancing interventions (Oberje et al., 2013; Simon-Tuval et al., 2016). Both reviews concluded that some studies showed promising findings with regard to cost-effectiveness, but that there is a need for more high-quality economic evaluations of adherence-enhancing interventions. We expect that our study will contribute to this evidence base. The finding in our study that the CATI intervention program is not cost-effective, is in line with previous studies that have shown that adherence-enhancing interventions are not cost-effective in comparison with usual care (Schroeder et al., 2005; Brunenberg et al., 2007). However, the systematic review by Simon-Tuval et al. (2016) concludes that community pharmacist interventions were either cost-saving or highly cost-effective. A possible explanation for the contrast between our study and this review is that only one of the studies included in their review targeted patients with hypertension. Although hypertension significantly increases the risk of cardiovascular disease (Lewington et al., 2002), symptoms are often mild or non-existent (World Health Organization, 2013). Thus, as compared to patients with other disorders, such as chronic obstructive pulmonary disease, hypertensive patients may feel less need for medication which in turn reduces adherence to the medication regime. In addition, only five studies were conducted in a Dutch setting of which three targeted participants with a chronic disease (hypertension, hypercholesterolemia, and ulcerative colitis). In the Netherlands, much effort has been put in multidisciplinary teams to improve the management of patients with chronic diseases (Schoen et al., 2006). It may be hard to improve on this further by the implementation of adherence-enhancing interventions.

The most plausible explanation for the lack of cost-effectiveness of the CATI intervention program is the choice for the MARS-5 to measure medication adherence. Although several studies have shown that the MARS-5 has acceptable validity and reliability (Cohen et al., 2009; Mora et al., 2011; Bäck et al., 2012; Salt et al., 2012; Horne, 2013; Lin et al., 2018), the cut-off value we used (MARS-5 <25) resulted in selection of participants with only marginally non-adherent behavior. A sensitivity analysis among participants with lower self-reported adherence at baseline (MARS-5 ≤23) shows more positive results confirming this potential explanation. Thus, it may be more efficient to target an intervention like this on patient groups with lower adherence. A possible explanation for the non-significant effect on quality of life is that health benefits of hypertension treatment become only visible after years of intensive treatment of hypertension. Finally, the CATI intervention program comprised only two consultations with the pharmacist. Although this reduces the costs of the intervention, this may not have been intensive enough to actually change participants’ behavior. Further research should clarify how and for whom more intensive adherence-enhancing interventions should be implemented to actually improve adherence and health outcomes.

Although total societal costs were not significantly different between groups, costs in all categories were non-significantly higher in the intervention group as compared to the control group. A potential explanation for this finding may be that there were substantially more participants with comorbid disorders in the intervention group than in the control group, although this difference was not statistically significant.

### Strengths and Limitations

There are several strengths and limitations of this study that need to be mentioned. The first strength is that this study was designed as a pragmatic RCT. A RCT is generally considered the most valid research design, because the risk of bias is minimized by the randomization procedure. Moreover, the pragmatic design ensures that the results of the economic evaluation can be used by health care decision makers. A second strength is that the study was conducted from a societal perspective, meaning that all relevant costs and effects were taken into account. Thus, not only health care costs were assessed, but also lost productivity costs. Moreover, a wide range of clinical outcomes was assessed including adherence and patients’ beliefs about medicines use, but also quality of life expressed in QALYs. QALYs are the outcome of primary interest in economic evaluations for health care decision makers, because the use of QALYs allows them to compare interventions across disorders and patient groups. A potential limitation of the study is the rate of missing data (20.6%). Although there were no differences in baseline characteristics between participants with and without complete follow-up, we cannot rule out that there was no selective dropout from the study. However, we tried to overcome this limitation by using multiple imputation, which is generally considered the most valid technique to deal with missing data (Sterne et al., 2009). Secondly, participants and pharmacists could not be blinded due to the nature of the intervention. Finally, the cost questionnaires had a recall period of 3 months which may have led to recall bias. However, studies have shown that participants can reliably report on health care utilization using questionnaires with structured closed questions for recall periods up to 6 months (van den Brink et al., 2005).

### Implications for Practice and Research

Based on the current study, we do not recommend the implementation of the CATI intervention program in the current form for the population selected in this study. Several adaptations to the CATI intervention program may be considered. First, the cut-off on the MARS-5 we used may have been too high resulting in inclusion of participants with only marginal medication non-adherence. Considering the more positive outcomes in the *post hoc* subgroup analysis on less adherent participants, future studies should focus on establishing the (cost-)effectiveness of interventions similar to the CATI intervention program in populations with lower adherence rates. Secondly, it is important that future studies also include even a longer term follow-up than in the current study, because health effects may only become visible after a long period of adequate treatment, meaning that economic benefits can probably be expected in the long term only.

## Author Contributions

JB coordinated and performed data analyses, reported study results, and drafted the manuscript. DvdL developed the study protocol, coordinated data collection, performed data analyses, reported study results and drafted the manuscript. YY collected data, performed data analyses, reported study results, and revised the manuscript. PE, CB, GN, and JH developed the study protocol and revised the manuscript. All authors read and approved the final manuscript.

## Conflict of Interest Statement

The authors declare that the research was conducted in the absence of any commercial or financial relationships that could be construed as a potential conflict of interest.

## Abbreviations

CATI: Cardiovascular medication non-Adherence Tailored Intervention
